# Editorial: The Oral Microbiome Is a Key Factor in Oral and Systemic Health

**DOI:** 10.3389/fmicb.2022.855668

**Published:** 2022-02-14

**Authors:** Denis Bourgeois, Lucio Souza Gonçalves, Josué da Costa Lima-Junior, Florence Carrouel

**Affiliations:** ^1^Health, Systemic, Process, UR4129 Research Unit, University Claude Bernard Lyon 1, University of Lyon, Lyon, France; ^2^Laboratory of Molecular Biology and Immunology, Postgraduate Program in Dentistry, Faculty of Dentistry, Estácio de Sá University, Rio de Janeiro, Brazil; ^3^Immunoparasitology Laboratory, Oswaldo Cruz Institute, Oswaldo Cruz Foundation, Rio de Janeiro, Brazil

**Keywords:** inflammation, saliva, microbiota, virus, biomarker, periodontitis, chronic disease, cancer

The oral cavity is a dynamic ecosystem comprising an assemblage of microbial communities, including many pathogenic or opportunistic species (Proctor and Relman, 2017). After the gut microbiome, the human oral microbiome (HOM) is the largest microbial community in the human body. The HOM plays a role in the onset and progression of several localized and systemic diseases including those of bacterial, viral and fungal origin (Soffritti et al.). In this Special Issue, there is a consensus: the oral microbiome is a key factor in oral and systemic health. Also, considering HOM as biomarkers for diseases is a significant emerging orientation. Saliva, the cornerstone of the HOM and systemic health relationship, has gained popularity as a readily available source of biomarkers useful for diagnosing specific oral and systemic conditions (Ferrari et al., 2021).

In oral health, biomarkers in saliva (e.g., enzymes, antibodies, protein markers, or oxidative stress markers) can be used for activity determination and for periodontal disease prognosis (Podzimek et al., 2016). The presence of key pathobionts and ongoing gingival inflammation are critical to the progression of periodontal disease. Mediators of periodontal disease initiation, progression, and recurrence are related to dysbiosis within the subgingival biofilm microbial community and the host immune response generated (Martínez et al., 2021). Abnormal changes in bacterial correlations, community structures, and local stability are linked to the dysbiosis observed in periodontal or peri-implant disease (Zhang et al.). Current findings indicate that salivary markers of oxidative stress are indicative of other clinical disease indices such as the papillary bleeding index and the caries index. Fungal species *Candida dubliniensis* and *Candida tropicalis* more abundant in the saliva of children with severe early childhood caries should play a role as caries risk markers (de Jesus et al.).

Recent studies have also demonstrated the role of periodontal disease as a risk factor or potentiator of distant systemic pathologies such as diabetes, inflammatory bowel diseases, Alzheimer's disease and oral cancer, further highlighting the importance of the oral cavity in systemic health (Kapila, 2021). This allowed for further characterization of oral microbial dysbiosis, especially putative bacterial periodontopathogens and changes in the composition of the oral virome during disease. New microorganisms (viruses, phages and bacteria) have recently been identified for their role in disease progression (Sedghi et al., 2021).

Thus, oral bacteria could be biomarkers for diseases such as specific cancer types. Some specific bacteria such as *Capnocytophaga gingivalis, Peptostreptococcus sp., Porphyromonas gingivalis, Prevotella sp*. and *Streptococcus sp*. are strongly associated with oral cancer (Karpiński, 2019). Bacteria belonging to genera *Actinomyces, Clostridium, Enterobacteriaceae, Fusobacterium, Haemophilus*, and *Veillonella* are linked to epithelial precursor lesions and oral cancer (La Rosa et al., 2020). Oral bacteria are detected in tumors outside the oral cavity. Every day, about 10^11^ bacteria from the oral cavity migrate into the lower gastrointestinal tract (DeClercq et al., 2021). Microorganisms act as chemical converters and metabolize nutrients from the host and from the diet (Anand et al., 2016). HOM can generate an ectopic colonization and produce numerous microbial metabolites capable of promoting tumorigenesis through the modulation of pathways related to energy homeostasis, immunological balance and nutritional intake (Zhang et al., 2016).

Oral pathobionts are essential in the development of colorectal and pancreatic cancer with current evidence showing differences in oral microbiota composition between patients with and without digestive cancers (Reitano et al., 2021). In cases of colorectal cancers, two periopathogenic species in particular have been frequently mentioned: Fusobacterium nucleatum and *Porphyromonas gingivalis*. In pancreatic cancers, in addition to previously mentioned bacteria, strains of *Aggregatibacter actinomycetemcomitans, Neisseria elongata*, and *Streptococcus mitis* have been described (Fan et al., 2018). Oral *Prevotella* species play an important role as commensals in health but can also be involved in diseases of the lower airways and upper gastrointestinal tract (Könönen and Gursoy). Oral bacteria from genera *Capnocytophaga* and *Veillonella* are apparently present in increased amounts in lung cancer patients (Yan et al., 2015). The study of complex interaction between the oral and gut microbiome in the pathogenies of type 1 diabetes is advanced, and suggests the use of saliva microbiome composition for early diagnosis (Moskovitz et al.). Also, bacterial dysbiosis plays an important role in the esophageal carcinogenesis process through microbial metabolism, inflammation and genotoxicity (Dan et al.) and *Capnocytophaga* and *Veillonella* are reportedly present in increased amounts in lung cancer patients (Najafi et al., 2021). At least, a predictive salivary microbiome signature is associated with a high risk of developing cardiovascular diseases (Murugesan et al.).

It is important for prevention of viral infection to draw a perspective on the role of the oral cavity in the virus infection (Tada and Senpuku, 2021). Salivary markers for viral infections involve direct detection of specific viral antigens, such as proteins and nucleic acids or host antibodies to viral infections and may provide a high accuracy point-of-care platform for detection of viral infections. HOM dysbiosis may facilitate inflammation and virus replication, limiting the development of a protective IgA response (Soffritti et al.). Immunity in saliva is, in particular, thought to have considerable impacts on the incidence and progression of respiratory viral infection. Parts of antiviral mechanisms against influenza virus and SARS-CoV-2 by immunity in saliva are similar.

Saliva based biomarkers are useful in diagnosis of several viral infections such as hepatitis A virus, hepatitis B virus, hepatitis C virus, Human immunodeficiency virus (HIV) 1, etc. (Zhang et al., 2016). Of course, several viruses have previously been isolated from saliva such as cytomegalovirus, Ebola virus, human herpes virus, herpes simplex virus, Influenza virus A, etc. (Corstjens et al., 2016). More recently, Zika and SARS-CoV-2 have been identified (To et al., 2020). In people living with HIV saliva might be used as a diagnostic tool for antioxidant changes in the future (Amjad et al., 2019). Oral bacterial species (e.g., *Leptotrichia spp*.), possessing unique niches and invasive properties, coexist with Human Papilloma Virus (HPV) within HPV-induced oral lesions in head and neck cancer patients (Mougeot et al.).

The fact that oral cavity is an important site for SARS-CoV-2 infection implicates saliva as a potential route of SARS-CoV-2 transmission (Carrouel et al.). The susceptibility of each individual to SARS-CoV-2 infection could therefore be characterized by HOM profile, which could facilitate virus replication and inflammation or conversely induce a protective IgA response (Soffritti et al.). Several routes of SARS-CoV-2 viral entry into the saliva have been suggested. There is direct entry to the oral cavity from upper and lower respiratory tract secretions, while circulatory viruses in the blood enter the gingival crevicular fluid. Studies reported a high yield of virus particles in the gingival sulcus and crevicular fluid, which are suspected to provide favorable conditions for virus replication and maintenance (Sri Santosh et al., 2020). Informations from the 2019 coronavirus pandemic highlight the link between oral and systemic health in a setting of viremias/bacteremias/microbemias, systemic inflammation, and/or immune system disruption in a susceptible host (Martínez et al., 2021).

Overall, the 12 contributions that make up this Special Issue highlight the fundamental relationship between HOM and systemic health providing potential microbiome-based clinical applications improving prevention, diagnosis, or drug response, which is of great significance. Characterization of microbial biomarkers is of great interest for precision medicine and represents a simple method to transfer microbiome research into clinical practice (Gilbert et al., 2018). If during the last years, an extraordinary effort has been made to identify biomarkers, today, HOM investigations have reached a critical inflection point. With the deepening understanding of the association between the HOM and other human microbiomes certain pathogens may be utilized as potential diagnostic biomarkers, screening tools, and prognostic indicators and interventions related to an altered human microbial composition may become the new adjuvant treatment in oral and systemic health.

## Author Contributions

All authors listed have made a substantial, direct, and intellectual contribution to the work and approved it for publication.

## Conflict of Interest

The authors declare that the research was conducted in the absence of any commercial or financial relationships that could be construed as a potential conflict of interest.

## Publisher's Note

All claims expressed in this article are solely those of the authors and do not necessarily represent those of their affiliated organizations, or those of the publisher, the editors and the reviewers. Any product that may be evaluated in this article, or claim that may be made by its manufacturer, is not guaranteed or endorsed by the publisher.
